# Providing End-of-Life Care to COVID-19 Patients: The Lived Experiences of ICU Nurses in the Philippines

**DOI:** 10.3390/ijerph191912953

**Published:** 2022-10-10

**Authors:** O-Jay B. Jimenez, Sheilla M. Trajera, Gregory S. Ching

**Affiliations:** 1 Graduate School, University of St. La Salle, Bacolod City 6100, Philippines; 2 Faculty, College of Nursing, University of St. La Salle, Bacolod City 6100, Philippines; 3 ICU Nurse, Bacolod Adventist Medical Center, Bacolod City 6100, Philippines; 4 Faculty, BSN, MN, and PhD Programs in Nursing, University of St. La Salle, Bacolod City 6100, Philippines; 5 Faculty, Graduate Institute of Educational Leadership and Development, Fu Jen Catholic University, New Taipei City 24205, Taiwan; 6 Research and Development Center for Physical Education, Health, and Information Technology, Fu Jen Catholic University, New Taipei City 24205, Taiwan

**Keywords:** palliative care, hospitals, intensive care units, nurses, COVID-19 pandemic

## Abstract

In the midst of COVID-19, radical change in the work environment further exacerbated the detrimental effects of critical illness in the intensive care unit (ICU). This may be heightened if the patient experiences a lamentable end-of-life experience due to inadequate end-of-life care (EoLC). Anchoring on the theory of bureaucratic caring and the peaceful end-of-life theory, insights can be gained into the motivations and behaviors that support the delivery of palliative care during COVID-19. With this having been having said, the objective of this study was to use a narrative approach to examine the lived experience of 12 nurses who provided EoLC in the COVID-19 ward of several hospitals in the Western Philippines. Participants’ narratives were transcribed, translated and analyzed. Among the themes that have emerged are: establishing a peaceful journey to death, holistic caring for the end of life, venturing into risky encounters in the call of duty, staying close amidst the reshaped work environment, and preparing the family life after a loved one’s departure. The study identified the importance of assisting patients on their journey to a peaceful death, but this journey was also accompanied by a sense of self-preservation and safety for colleagues and families.

## 1. Introduction

A patient’s quality of life is already compromised by critical illness if they are in the intensive care unit (ICU), which can be exacerbated by the appalling quality of death and indignity associated with poor end-of-life care (EoLC) [1]. Prior to the pandemic, ICU nurses played a key role in providing care to critically ill patients by utilizing a variety of technologies, assessing and managing complex disease conditions, and providing appropriate care with respect for other health care professionals [2]. This vital role involves a wide range of clinical settings, including the care of patients with serious illnesses and integrating their families while advocating for quality EoLC [3]. These situations put nurses in a precarious position to make decisions for patients who are more often unable to make the decisions for themselves, resulting in adverse effects for ICU nurses, such as emotional and psychological burnout and reduced job satisfaction [4,5]. 

For more than two years, COVID-19 has been impacting ICUs in the Philippines with cases that are highly prevalent and morbid. Patients frequently present with worsening symptoms and an elevated need for palliative, supportive, and EoLC [6]. Moreover, with the constant fear of becoming infected, those in the profession have become very stressed [7,8,9,10]. It is evident that the way ICU nurses work has changed in the context of the pandemic [11,12]. Without a doubt, the efforts of nurses to provide life-saving procedures during COVID-19 are very commendable [13,14]. However, limited information is still available on the experiences of COVID-19 ICU nurses working in the Philippines in relation to the EoLC process.

In the midst of the COVID-19 outbreak, hospital protocols have radically changed, presenting multiple obstacles to quality EoLC [15,16]. For example, family members are not allowed to stay and be physically close to patients, while nurses assigned to COVID-19 patients in ICUs have many restrictions. Additionally, discussions at EoLC family conferences have become more difficult as family members are restricted to virtual meetings. These situations altogether add up to the vulnerability of the mental and physical health of ICU nurses [17]. Against this background, it is therefore important to identify aspects of care and implementation obstacles that are essential for the care of critically ill ICU patients at the end of their lives. In order to achieve this, it would be worthwhile to first understand and analyze the key experiences of ICU nurses in this area. In particular, it is important to undertake a study c in the Western Philippines, where this type of study is relatively scarce.

The current study is based on the concepts of the theory of bureaucratic caring [18] and the peaceful end-of-life theory [19]. These theories provide insights into the purpose and behavior that support EoLC provision without protocols in place. Based on the theory of bureaucratic caring, the provision of care is emphasized within organizations. Caring may be characterized by its dynamic relationships between right action and charity, between love and compassion expressed in response to suffering or need, and by fairness or justice regarding on what should be done [20]. Having an understanding of how human beings, environments, and circumstances are interconnected is fundamental to a better understanding of this type of theory. In addition, it provides a specialized perspective on how healthcare institutions, healthcare systems, organizational bureaucracies, and nursing care are integrated and coordinated with each other, as wholes and components in the health and organizational system [21].

On the other hand, the peaceful end of life theory focuses on the structural setting, that is, the family system, which includes both the terminally ill patient and all other family members who are being cared for in an acute care setting. Quality of life is therefore seen as an essential concept in providing high quality EoLC [22,23]. Ruland and Moore [19] assume that feelings and events during the end of life experience are individualized and personal. The goal of EoLC is not to optimize the care provided, but rather to maximize treatment that includes the best possible care, including technological and comfortable measures to improve and achieve quality EoLC [24]. Suffering outside of physical ailments is not readily understood, but alleviating suffering is a fundamental goal of EoLC and is necessary to achieve comfort and a peaceful end of life experience. 

In these context, a quality EoLC can be considered an invaluable aspect for critical COVID-19 patients. Undoubtedly, COVID-19 has brought fear and pain not only to patients but also to nurses in the ICU [25,26]. In addition to the stress and fear of contracting the virus [27], nurses’ accounts from previous lived experience studies describe their unrelenting efforts to provide comfort and care to their patients [28,29,30]. Furthermore, nurses too are required to use a variety of coping mechanisms to cope with difficult times [31,32], whether they are religious oriented [33] or counselling among peers [34,35]. More importantly, amidst all of these factors, nurses are expected to be resilient as the work paradigm changes [36,37], while maintaining close communication with family members of the patients [38]. 

Both the theory of bureaucratic caring and peaceful end of life theory provided fundamental support for the importance of ensuring that the patient achieves a comfortable, dignified, and peaceful end of life experience, a peaceful end of life requires complex and holistic nursing care. These theories provided the basis for explaining the various untold experiences encountered by ICU nurses during the COVID-19 pandemic as various factors influence the spiritual and ethical care of dying patients.

## 2. Materials and Methods

### 2.1. Study Design

This study was designed within a narrative perspective in which ICU nurses provide personal accounts or stories reflecting their experiences with COVID-19 patients during EoLC [39]. It also presents the sum of the experiences of several individuals who have all undergone the same experience. Due to the COVID-19 pandemic, the term lived experience is frequently used to describe the journey that nurses undertake [40,41]. This translates into a desire to understand how a person’s experience, in this case the experience of the ICU nurse, is absorbed into their awareness. Consequently, this also translates into a desire to understand the significance of such experience.

### 2.2. Participants

Study participants were twelve (12) carefully selected ICU nurses deployed to COVID-19 units from multiple hospitals in the Western Visayas region of the Philippines. The participants were chosen based on assurances that they hold relevant experience and possess a genuine understanding and viewpoint of the phenomenon being considered [42]. Inclusion criteria include: currently employed as an ICU nurse, at least six (6) months experience as a nurse in the ICU, at least one (1) month of experience in the COVID-19 critical care area in the hospital, has experienced providing EoLC to a critically ill or dying COVID-19 patient that may or may not be on do-not-resuscitate (DNR) status, within the age range of 25 to 45 years old, either male or female, holds at least a bachelor’s degree, either single or married, works in either a private or public (government/state) hospital, and has knowledge of providing EoLC.

Table 1 shows the participants’ backgrounds, career descriptions, and pseudonyms. Of the 12 participants, eight were male and four were female. The average age was 34 years old. Eight work in private hospitals, while the remaining four work in state or public hospitals. The average length of service as a nurse is nine years, with all participants having at least three years of ICU experience. In terms of experience with COVID-19 patients in the ICU, the majority of participants had more than five months of experience, whereas two participants had one and two months of experience, respectively.

### 2.3. Data Collection and Analysis

Recruitment of participants began after the study protocol was reviewed and approved by the University of St La Salle Graduate Program review panel and ethics committee. The participants were initially recruited using the purposive sampling method guided by the aforementioned inclusion criteria. To bolster the recruitment of the participants, the snowballing technique, in which hospital contacts recommended participants who met the research criteria and who might also be willing to participate in the study [43], was used. This method was selected specifically for its ability to reach hard-to-find populations [44]. The final sample size of 12 participants was set once saturation was reached or no new concepts or themes emerged during the interviews [41]. 

Potential ICU nurses assigned to different hospitals were contacted and invited through Facebook Messenger, e-mail, or mobile phones. As part of the informed consent process, all information about the study was disclosed to the participants. The study was initiated by sending a letter of request and an informed consent form which included information about the study, such as the introduction and purpose, participant selection, a clause for voluntary participation, procedures, risks and benefits, and a confidentiality agreement.

Once consent was obtained, a schedule for the interview was arranged. Participants were free to choose which method of online communication was most convenient for them (Zoom, Facebook Messenger, Google Meet, Skype). The interview was scheduled to last for 30 min to an hour. Participants were also encouraged to select a convenient time and location that would enable them to be comfortable, relaxed, and at ease during the interview. In view of the ongoing COVID-19 pandemic, the participants were initially offered the option of conducting the interview online. However, if the participant decided to conduct the interview face-to-face, precautions were taken accordingly. In total, only two participants chose to conduct the interview face-to-face, while the remaining ten chose online interviews. Prior to the interview, participants were reminded that the session would be recorded and that they may stop the interview at any time. All interviews were conducted in English. However, participants were free to use any language they wished to share their thoughts. In the later transcription of the data, non-English languages were carefully translated into English. Participants were also asked to select their preferred pseudonym to be used for the transcription. After the transcription was completed, the participants were asked to validate the accuracy of the data gathered.

A description of the ICU nurse’s experience was derived and acquired through the use of narratives or stories about their lived experience during their involvement in the caring of the dying COVID-19 patient. A guiding context was provided to encourage participants to share more about key moments in the provision of EoLC. For instance, an overarching question was asked: “What is your experience of providing EoLC to a dying COVID-19 patient?”. Second, several preset probing questions were also used to help confirm and clarify certain descriptions or statements made by the participants. Examples of probing questions were: “What are the different things you do while providing EoLC?”, “What was the patient’s response like while receiving EoLC?”, and “Is there anything else that you would like to discuss in relation to your experiences in EoLC?”. The content of the guide also enabled the determination of the profile of the participants, which included their age, gender, marital status, educational level, length of service, hospital type (whether private or public), hospital location, and the number of months being assigned to a COVID-19 critical care unit.

Data analysis followed Colaizzi’s [45] method. This approach provides a rigorous analysis through a unique seven-step process in which each step remains close to the data in order to ensure the credibility and reliability of the results, as well as exposing emerging themes and their interwoven relationships [46]. This approach depends on the rich first-person accounts of experience that may emerge from interviews conducted online or face-to-face [47]. The seven steps in Colaizzi’s [45] descriptive method include the following: (1) familiarization by reading and re-reading the ICU nurses’ narrative transcripts multiple times, (2) identifying and extracting significant statements from the participants that relate to their profound experience while providing EoLC, (3) forming meanings from the statements of the participants, (4) grouping and organizing the ICU nurses statements to form relevant and meaningful themes, (5) developing an exhaustive description of the thematic findings, (6) constructing the basic structure, and (7) seeking verification of the basic structure and to validate the results by returning them to the participants and confirming the results with their experiences [45]. 

Experiences and thematic statements of the participants were highlighted in order to generate a series of themes as to how ICU nurses experience EoLC on a day-to-day basis. The intuitive process was completed by establishing rapport and contact with the participants, paying particular attention to their daily activities and absorbing their lifestyle. Intuition refers to the researchers’ attempt to empathize with the situation by empathizing with a feeling or thought of the participants that they have experienced themselves. This occurs when researchers remain open to the meanings attributed to the phenomenon by those who have experienced it [48].

## 3. Results and Discussions

From the narratives of the participants, five main themes were identified that are consistent with both the theory of bureaucratic caring and the peaceful end of life theory: establishing a peaceful journey to death, holistic caring for the end of life, venturing into risky encounters in the call of duty, staying close amidst the reshaped work environment, and preparing the family life after a loved one’s departure.

### 3.1. Theme I: Establishing a Peaceful Journey to Death

Patients with COVID-19 infection and the novelty of this disease pose a complex set of complications, particularly in patients with co-morbidities and the unvaccinated [49]. In caring for the dying COVID-19 patients, the primary goal of most participants was to ensure that the patients could have a peaceful death at the end of their lives. Several instances were identified in the narratives, reflecting the participants’ experience in delivering EoLC. In addition, the results also show the various core components that form a cluster of aspects that ensure a peaceful end of life to dying COVID-19 patients. 

For instance, participants have shared their perceptions on what a peaceful death looks like. Geoffrey mentioned: “*… they already consent that they will not move to the aggressive side, because they already accept the situation; they know that the end is actually death, so probably that would be one thing where I could really say that the patient died peacefully.*” This means that acceptance is not only on the part of the patient, but also by the relatives who allow the patient to go to the afterlife with no baggage, which can be symbolized through advance directives or DNR forms. With these vital consents, the patients are allowed to move on peacefully, unlike those with no DNR, as stated by Siegfried: “*I could really see and say that the patient was really at peace if they are on DNR status, because they become flat on their own.*”

Importantly, although the global pandemic has already claimed the lives of millions of people, ICU nurses have retained an innate desire to help patients end their lives in comfort by providing EoLC. These efforts can be seen through the three emerging sub-themes: witnessing acceptance of mortality and the inevitability of the end of life, assisting in a symptom-free death and the alleviation of suffering, and fostering a graceful and dignified death.

#### 3.1.1. Witnessing Acceptance of Mortality and Inevitability of End of Life

This is relative to ICU nurses who witnessed dying COVID-19 patients say “yes” to death. When death is due to a natural cause associated with old age and there is an ability to make meaning of death, there is a neutral acceptance of the end of life and mortality [26]. The participants asserted that patients who were able to accept their eventual voyage to the afterlife were able to verbalize appreciation and actual acceptance when their spirituality and wellbeing were addressed. Acceptance is not only widespread among patients, but also among their loved ones, as they religiously elevate their lives to God when inner burdens are removed and problems with loved ones are resolved. Participants shared how spiritual enlightenment helped their patients achieve peace. Shiloh mentioned: “*No matter how toxic the patient is or how grumpy the people are; they are spiritually enlightened about things that are happening.*”

Most participants believed that when treating and healing critical COVID-19 patients is already a lost battle, giving comfort and peace is the highest priority. Witnessing acceptance of inevitable death comes in various forms, such as verbalizing, as stated by Winifred: “*She (patient) said thank you, grateful that she could speak to the chaplain before she died. So her spiritual being is complete. She was able to speak. Then we talked about what she felt…*” Another form is when the patient or the family of the comatose patient signs an advance directive, as stated by Geoffrey: “*The patient has advance directives…, especially if the patient himself signed the advance directives, so, probably, if everything else is exhausted…, let’s say his own breathing, his own mechanism, his own heartbeat; probably I could say that he died peacefully, because that’s his choice…*” Essentially, the participants believed they had experienced how they had helped the patient through various interventions with empathy and caring.

#### 3.1.2. Assisting in Symptom-Free Dying and the Alleviation of Suffering

In the course of the disease, dying COVID-19 patients have displayed diverse types of symptoms including fever, cough, difficulty breathing, and pneumonia-related symptoms, as well as abdominal pain, chest pain, and headaches [25]. Severe symptoms are one of the main causes of suffering in dying COVID-19 patients. It is the anguish caused by the experiences perceived by the patients’ loved ones that drives them to take their patient out of suffering and sign the DNR forms. Callum explained: “*Occasionally, family members decide against intubation because they do not wish to witness their family member suffer further and add to the agony of the patient.*”

The narratives also disclosed various methods for managing symptoms and pain; for instance, pharmacologic approaches through pain medication and sedation for patients. Solomon noted that: “*So, in the end, I hope that my support will somehow ease their suffering. Especially the family members. Sedation is the most effective measure to alleviate the patient’s suffering. It is better to die comfortably than in agony.*” A participant indicated that a doctor stated that sedation may also cause a slight degree of amnesia, so that the procedure will not be as traumatic for the patient. This was affirmed by the statement of Serena when she said: “*Being sedated also makes you feel as if you are experiencing amnesia. Therefore, you will not remember the time that you were intubated, …as being intubated and in the hospital is a very traumatic experience.*” Consequently, it is imperative that different methods be attempted for managing symptoms and pain. Studies have shown that even the use of warm blankets can reduce agitation, pain, and the use of analgesics. Additionally, displaying visually appealing elements such as family photographs or natural scenery may encourage patients to relax [28]. 

#### 3.1.3. Fostering a Graceful and Dignified Death

According to findings, participants in either public or private hospitals continue to be committed to ensuring a dignified death. From the participants’ experiences, safeguarding privacy, respect, and confidentiality preserves the dignity of the dying. This involves dressing the patients, particularly the female patients, preventing exposure of the private parts and utilizing blankets to conceal their bodies. The integrity and dignity of the patient are maintained regardless of whether the patient is conscious or not. Solomon affirmed this by stating: “*Although a patient is dying, you continue to provide them with nursing care. I give the same level of care as I would to other patients who are not terminal. Nothing has changed. Both dying and non-dying patients receive the same care…*”.

Nurses who specialize in ICUs provide post-mortem care to patients not only during the dying process, but also after the patient dies. Solomon added: “*Even when they were on the verge of death, our daily care ensured that at least they felt that they were not worthless,*” and “*…that they are still human beings. Even if they have a very poor prognosis, they are still effectively treated…*” as expressed by Irene. Important here is that ICU nurses are cognizant of the reality of death and commit themselves to making it as painless, comfortable, and dignified as possible, despite the environment of critical care that encourages a paradigm of curing rather than one of caring [29]. 

Nurses serve as an invaluable substitute for relatives who cannot be with their patients. In providing the patients with the assurance that they are not alone, the nurse also safeguards the patients’ privacy and integrity, ensuring that they die in dignity [30]. In addition, participants emphasized that maintaining dignity for dying COVID-19 patients should extend throughout the dying process and into the final moments of the patient’s life in the ICU. Milo revealed: “*While the Glasgow Coma Scale (GCS) score of the patient is 3 and the tube has already been removed, you should position the patient correctly and clean them if needed. At least the patient retains their dignity until the end of their life. Ensure that they are still presentable when they pass away*.”

In sum, ICU nurses strive to minimize the anxiety and discomfort of dying patients. Moreover, ICU nurses believe that dying patients should have some control over how and with whom they spend their last moments. Importantly, despite the presence of a crisis standard of care within which the healthcare team and nurses are operating, it is vital that patients continue to receive compassionate EoLC [50]. According to the theory of bureaucratic caring, this compassionate EoLC represents the concept of caring. As noted earlier, the concept of caring may be seen as a dynamic relationship between service and correct action. When caring for dying patients, it is important to ensure a dignified death, which includes respecting their wishes and ensuring their comfort. When it comes to the care of patients at the end of life, the primary goal must be to eliminate all pain and fear.

### 3.2. Theme II: Holistic Caring for the End of Life

The second theme relates to the participants’ perceptions of patient needs and how they provide holistic EoLC to their dying COVID-19 patients. Specifically, these interventions are categorized into perception of mental support, physical support through nursing interventions, emotional support, spiritual care, and social support. These experiences were confirmed by Ray’s theory of bureaucratic caring, which discusses how nursing is a spiritual, relational, ethical, and holistic profession that seeks the good of both self and others [18,20]. According to Serena, “*I made sure I provided a holistic approach since I believe there are four levels to holistic care within a patient’s life: physical, emotional, social, and spiritual.*” For the participants, the physical, emotional, social, and spiritual care and support are all interconnected.

Even during COVID-19, the implementation of holistic EoLC is still possible. Milo added that, “*…despite the pandemic, we continue to provide proper care. As far as feeding and suctioning are concerned, they remain the same. We continue to clean the patient, bathe the patient, and provide holistic care to the patient*.” Furthermore, these statements demonstrated that a holistic approach leads to not only a peaceful death, but also a dignified one. Geoffrey reiterated, “*…throughout the end of life journey how meeting the patient’s needs equates to a peaceful death. In my opinion, it could be considered decent dying. If you were able to address the concerns of the patient holistically, then you may consider it; the dying process is actually decent.*”

There may have been differences in hospital policies (between private and public institutions). In spite of this, nurses retain their ability to care for patients and address their needs in a holistic manner. Even with the fear of contracting COVID-19, and the limited time spent at the patient’s bedside, the statements of the participants appear consistent in their approach to the holistic care of the dying. Furthermore, despite the fact that COVID-19 patients are dying and have a poor prognosis, holistic care may continue to influence their mental health and satisfaction [51]; hence, it is still critical to emphasize the individualized aspect of care. Several sub-themes were identified from the clusters of statements: the shift from a negative to a positive outlook on death, giving relentless bedside care for physical needs, lifting burdened emotions over adversity regardless of patient’s response and consciousness, instilling faith to embrace fate, and surrounding by the presence of the intangible loved ones.

#### 3.2.1. The Shift from a Negative to a Positive Outlook on Death

In this sub-theme, participants described different interventions provided to dying COVID-19 patients with regard to their mental health. Considering the proliferation of information regarding COVID-19’s horrific effects on many lives, participants experienced encounters with dying patients who already knew that their death was imminent. Patients are mostly alone in their respective rooms and experience mental difficulties as a result of their knowledge of their condition. During the course of the study, the participants witnessed patients going through the stages of denial, anger, bargaining, depression, and acceptance (DABDA). As Milo noted, “*They have certainly reached the point where they already know that they are dying and they could not survive this condition*.”

Axel added, “*Knowing that they are on the brink of death, their struggle is intense*.” Despite their own physical and mental struggles, ICU nurses still attempt to shift the perspective of dying COVID-19 patients. A perspective shift is the process of switching from your normal or usual viewpoint in order to discover new ways of thinking and understanding. Furthermore, in spite of the limited amount of time allowed inside the ICU, some participants attempt to converse with the patients and to provide them with comfort, especially if they are conscious. One of the participants, Serena, has shared that she “*sometimes shares some happy experiences with the patients so that they can at least try to imagine the world outside, or perhaps even imagine a better life for themselves*. *It is critical to maintain a positive attitude. At the same time, try to impart a positive outlook on them.*”

#### 3.2.2. Giving Relentless Bedside Care for Physical Needs

In the second sub-theme, participants describe their experiences performing different procedures to address the physical needs of patients during EoLC. Despite being critically ill and having a poor prognosis, the patient was consistently and timely fed, whether by tube feeding or parental feeding. The following findings illustrate how the physical support aspect of holistic care was implemented, despite the fact that death is an inevitability. As part of this process, bedside procedures such as suctioning, hygiene, bathing, and turning are performed. The routine procedures integrated into the EoLC of the dying patient will continue in the absence of a waiver or directive of DNR regarding the holding or refusal of medications, laboratories, or procedures in order to maintain the patient’s comfort. 

In the ICU, nurses persevere until the patient passes away on their own. According to Geoffrey, “*…we wish to address the patient’s concerns holistically as much as possible. In such a case, we should maintain the patient’s care, coordinate with the doctor, provide good nutrition, monitor the patient’s vital signs, administer available medications.*” Since the pandemic began, frontline healthcare workers have been working tirelessly. Having a relentless mindset is what allows you to continue to survive and overcome even when others are unable to do so [52]. Patients were provided with physical care regardless of their status or prognosis. According to Siegfried, “*No matter how busy, we always make sure that the medicine is delivered on time.*”

Relentless bedside care also includes providing comfort through touch and voice. It involves gestures of comfort provided by the participants while caring for the dying COVID-19 patients. Providing comfort, care, and solace to dying patients and their families is an important role nurses play, as they help them accept and cope with death [53]. Additionally, the voice of comfort on the deathbed provides reassurance to the patient that they are still being cared for, regardless of whether or not they are responsive. Furthermore, the soothing touches and prayers of participants can also help ease discomfort. Serena stated, “*This prayer provides relief and comfort to both our patient and the family... Even holding the hand will bring comfort to the patient, making sure they are not alone in this world.*” Touch plays an integral role in caregiving, which has been proven to reduce pain in patients. The peaceful end-of-life theory holds that feelings and events experienced at the end of life are personal and individualized; the gesture of “touch”, in this case, facilitates this connection [19].

#### 3.2.3. Lifting Burdened Emotions over Adversity Regardless of Patient’s Response and Consciousness

The third sub-theme pertains to the findings regarding the participants’ ability to provide emotional support to patients who are burdened with emotions. A common practice among ICU nurses was to maintain professionalism and empathy by putting themselves in their patients’ shoes. As Milo stated, “*It is essential to remain professional while also being empathetic to them and continue to provide them with care, meet their needs, and provide them with emotional support.*” Participants maintained communication with dying COVID-19 patients while they were conscious, responsive, or comatose without responding. It was important for the participants not to show any signs of weakness or distress as they understood that the patients were already emotionally exhausted.

According to Winifred, “*…talking to dying COVID-19 patients will help lessen their pain and divert their attention from the pain they are experiencing*.” Patients are also reassured that their families are doing everything possible for them and that they should follow the doctors’ recommended regimen. Irene shared, “*We reassure the patients that their family is doing all they can to help.*” The goal is to give patients a sense of security, knowing that their family cares about them and will not leave them alone. Along with providing emotional support through communication, a common experience for the participants is therapeutic touch. Using the hands that bring tranquility was an effective technique of providing emotional support and providing the dying patients with the sense that they were not alone. Solomon stated that he sometimes “*asks the family member which is the patient’s favorite song, then simply asks them to play that music.*” It is evident that lifting the emotional burdens of the patient is essential, regardless of their level of consciousness. It is critical to communicate with the patients and remind them that while these events are beyond their control, they can still learn to shift their reactions and emotions [54]. 

#### 3.2.4. Instilling Faith to Embrace Fate

In this sub-theme, participants discussed how they provided spiritual care to their dying COVID-19 patients. The pandemic has affected the way in which ICU nurses perform their duties, and this has not spared pastoral or spiritual care for the patient. All participants agree that it is crucial to pray for and with the patient. Milo stated that “*saying a prayer for them will always make them feel strong and hopeful.*” However, Geoffrey emphasized that before providing this care, “*we should first verify their religious beliefs and practices.*” Despite knowing the outcome of the patient’s journey, the participants still made sure that the patient received prayer to the full extent of their abilities.

Another challenge was the muffled sound of the prayer behind the mask and personal protective equipment (or PPE); the participants, however, still pray with the patient. It is believed among participants that the last sense to disappear is the sense of hearing, therefore spiritual care is extended to even the comatose. Because of strict COVID-19 health protocols, the participants resorted to virtual services like Zoom or other online platforms. They also give the patients some religious items like a rosary or a Bible. Siegfried said, “*For others, they just use a bible and a rosary. We give it to the patient. Then, for other patients who are already unconscious, they would use a cellphone with a recording that replaces prayer. So we just play and let the patient listen.*”

In order to die peacefully, patients may wish to reexamine and reiterate their beliefs. This is because the end of life is often the time when spiritual matters are brought to the forefront. Most religions emphasize living purposefully, involving submission to a divine entity and offering rituals that provide comfort and influence to patients and their families during their final days. As the pandemic unfolds, participants acknowledge the role of family and religious leaders in fostering spiritual renewal and meaning [33] within the provision of quality EoLC.

#### 3.2.5. Surrounding by the Presence of the Intangible Loved Ones

In this sub-theme, participants discussed methods of facilitating the continuous presence of dying patients’ loved ones via virtual means. During COVID-19, family members can only communicate using cellular phones, video calls online, or digital platforms; physical contact is not possible. Participants reported that their tangible presence was helpful in comforting the patient. Facilitating human contact and emotional connection between the family and the patient provided an invaluable source of comfort. Olivia stated, “*They are thankful that we helped them connect with their loved ones.*” The participants also provided heartwarming items such as pictures and cards with messages in order to increase the patients’ well-being. During significant occasions, the participants felt they represented the families of the patients. As Axel mentioned, “*I believe it was the birthday of one of the patients. To celebrate, we placed colorful balloons inside the room. Although I cannot recall if flowers were given, there are balloons in the room. We decorate the whiteboard and write Happy Birthday on it.*”

Due to the absence of a loved one, the participants served as the primary social support for the dying COVID-19 patients. According to Milo, “*I have also had experiences in which the patient was already in a critical condition. Even though he was unable to hear me, I continued to speak to him. I told him stories. I just continued to talk to him.*” In fact, the presence of a significant other, even with the use of virtual platform, has been shown to mitigate the effects of stress [31]. Even if the significant others are not tangible, their virtual presence through video or audio calls can help support the struggling COVID-19 patients. In other words, connecting to immediate family members is critical to delivering quality EoLC [19].

### 3.3. Theme III: Venturing into Risky Encounters in the Call of Duty

Theme III consists of a cluster of statements related to the participants’ perceptions of their role, their decisions, their feelings, their purpose, and their mission during their experience of providing EoLC to a dying COVID-19 patient. The following statements describe the events that occurred within the ICU in relation to the provision of lifesaving measures and EoLC by healthcare workers. According to the participants, they were fully aware of the dangers associated with their occupation in the course of performing their duties. There was a high level of caution among the participants due to the highly transmissible nature of COVID-19, and each encounter with a COVID-19 positive patient posed a risk. In reality, the perception of people’s own risk of contracting the disease increased significantly during the COVID-19 pandemic [9]. 

Participants described what it was like to work in the COVID-19 ICU during the outbreak of the COVID-19 pandemic. Siegfried explained, “*First of all, there is fear in the COVID-19 ICU. There were a lot of deaths caused by COVID-19. We didn’t know the proper safety precautions, such as PPE and double masking*.” Additionally, the participants described what the infected patients may breathe out in the form of droplets and minute particles containing the virus that other people may breathe in. There is a possibility that these particles may land on the eyes, mouth, or nose, as well as contaminate surfaces.

The participants in their statements shared that somehow, PPE made it more difficult to care for the dying COVID-19 patients. Callum explained, “*Wearing PPE is also a challenge. As a result of aerosols being used to transport viruses, we must wear full PPE, especially when working on an intubated patient.*” In spite of the danger involved, the participants believed they were obligated to perform their professional duty due to moral and personal obligations. Participant Olivia stated, “*That’s really a part of your oath when you became a nurse; so even if you’re not well compensated, you’re still happy. Happy with the profession that you’ve chosen.*” Overall, these perceptions, roles, feelings, and choices associated with the perilous duty were revealed in the following sub-themes: the dilemma between self-preservation and precarious interventions, spotting the signs of death, sensitivity to responses after breaking the news, elucidating conflicting emotions in desolating frontlines, and giving the best effort in everything despite the perceptions of futile care.

#### 3.3.1. Dilemma between Self-Preservation and Precarious Interventions

According to the first sub-theme, participants were faced with a dilemma as they had to choose between two options, each of which had undesirable outcomes. There have been instances in which participants have been torn between fear and the desire to maintain personal safety by wearing PPE. This can affect the speed with which they can respond to an emergency, which can lead to a worsening of the patient’s condition. Milo mentioned, “*There are times that we get carried away by our emotions; sometimes, we forget to protect ourselves, especially during the pandemic. At first, that is what we are saying, that there is no emergency in a pandemic. However, still, we are nurses, we have this caring nature, that even though we know that the patient is already in an emergency, sometimes, these are the moments that we commit mistakes, that we get infected, because of the procedures we perform; because we rush to aid our patient.*”

Participants indicated that wearing PPE was both physically and mentally taxing, as they needed to be sure they were well protected and sealed before entering the room. However, concerns remain about the possibility of viruses escaping or entering through leaks in the PPE. In addition, participants reported having ambivalence regarding the use of aerosolizing procedures such as suction and bag-valve-mask ventilation, which may improve the oxygenation of the patient, yet increase the risk of virus transmission. A dilemma is created by this situation since if they do not perform these interventions, the patient will deteriorate due to hypoxia, but if they do, they will increase the risk of aerosolizing COVID-19 and increasing its transmission. As explained by Irene, “*Back tapping is not permitted because we do not wish to stimulate coughing, especially if they are intubated*. *Maybe we can simply perform a close suction so that at least we can assist the patient.*”

Despite the fear of acquiring the virus through suctioning, Axel further confirmed the caring nature of ICU nurses by asserting the necessity to use the close-suction system to support the patients’ airway. As he mentioned, “*We secured and looked for a closed-suction system in order to support his airway, because it is quite difficult for nurses to perform suctioning, especially for COVID-19 patients. It was actually very risky on our part*.” In essence, despite the dilemma faced by COVID-19 ICU nurses, the testimonies of the participants still showed how fears for their personal safety are being overcome.

#### 3.3.2. Spotting the Signs of Death

The second sub-theme relates to statements by participants describing their role in providing EoLC to dying patients. ICU nurses are responsible for closely monitoring patients so that any signs of deterioration or abrupt changes in their condition may require immediate intervention to save their lives. For dying COVID-19 patients, EoLC requires a skill that permits them to recognize signs of impending death and to alert their physician and family, thus preventing untoward reactions such as shock, hysteria, and retaliation due to the fact that they were only informed after the patient’s death.

The participants observed that co-morbidities make it more difficult to wean dying COVID-19 patients off ventilators. According to Siegfried, “*most of the patients I have handled who have succumbed to COVID-19 also had co-morbidities.*” In addition, he added that “*…diabetic patients are more likely to suffer from kidney failure, which makes it impossible to wean them off their ventilator.*” Yet, despite their skills, participants were able to identify the signs that indicate that their patients might not survive. One participant, Irene, stated that “*of course, we are the ones responsible for holding their lives, but there are times when we feel that their time is coming. At times, we may feel that they are nearing the end of their lives. The signs are there.*”

In addition, most participants reported renal symptoms such as a decrease in urine output, laboratory findings such as acidotic ABG results, diagnostic procedures such as chest X-rays, and the mottled appearance of extremities. Detecting near-death events is one of the primary responsibilities of COVID-19 ICU nurses, since they are the ones who spend most of their time at the bedside, and they serve as a liaison between the patient and his or her physician. It has also been suggested that DNR status and advance directives may affect the course of dying of COVID-19 patients, as explained by Geoffrey, “*So those are the things; the only difference between the patient who has an advance directive and the patient who is still aggressive is that we can intervene.*” However, he added, “*if despite interventions, the patient continues to deteriorate when it comes to vital signs, then perhaps that marks the end of our patient’s life.*” 

#### 3.3.3. Sensitivity to Responses after Breaking the News

In this sub-theme, participants are portrayed as representatives of the healthcare team and of patients’ significant others. It is considered that the participants are the bearers of both blissful and horrendous news to the patient. Although some participants mentioned being proactive regarding the patient’s status in order to avoid inflicting distress on them, they should never forget that veracity prevails over all other considerations. Healthcare workers often lack the skills needed to effectively deliver bad news to patients and their families in the clinical setting.

Axel described, “*So after being told that they were about to die... It was almost as if they were begging for the doctors to prolong their lives.*” Although physicians commonly deliver bad news to patients, nurses also play a crucial role in doing so. Consequently, one should be trained in clinical and communication skills; to be able to convey bad news appropriately and effectively. With COVID-19’s rapid deterioration, ICU nurses often carry the burden of being listeners or witnesses to patients who can express their feelings when they hear the bad news.

Some patients undergo DABDA even after initially denying that they are near death. Participants have acknowledged the importance of gently informing the patient of their current status, which will eventually lead to acceptance. According to Milo, “*…you should explain it slowly; first explain it in medical terms, then explain it in layman’s terms. There is no question that the participants need to understand what the treatment is or what course of treatment will be taken and what the next steps will be for them.*” The participants understood the need to be professional and to empathize with the dying patients. 

#### 3.3.4. Elucidating Conflicting Emotions in Desolating Frontlines

In the course of an epidemic outbreak, both negative and positive emotions are experienced by the front-line nurses. A negative emotion was dominant at the beginning, followed by a gradual development of a positive emotion. In reality, maintaining the mental health of nurses depends on self-coping styles and psychological growth [32]. The everyday life of COVID-19 nurses is described as being filled with mixed emotions. In spite of this, it is very important to maintain the appropriate level of composure [10]. Milo stated, “*Whenever possible, we should not demonstrate any weaknesses; we should not show any signs of grief as this may be transmitted to the patient.*” Another burden that accompanied their struggles was the fear of exposure and the transmission of COVID-19. Axel explained, “*Nurses who care for COVID-19 patients are heavily burdened. In order to protect ourselves, we had to wear PPE at all times, which limited our ability to move and function... I am no longer able to handle patients as effectively as I once did.*”

#### 3.3.5. Giving the Best Effort in Everything despite the Perceptions of Futile Care

In the course of the discussion, participants acknowledged that they were already providing futile care to patients with poor prognoses. Despite their best efforts, they knew that they would not be able to protect everyone from this epidemic [37]. Serena commented, “*It is heartbreaking for a nurse to witness this. No matter how much we would like to save our patients, it is impossible to save them all. Thus, what we do is to provide them with the best possible care during their final stages of life.*” Additionally, participants believed they had already maximized everything before the patient died, giving them the satisfaction of knowing they had done their best. According to Shiloh, “*we could not blame ourselves, because we did everything in our power to keep the patients alive.*” The participants provided EoLC to these patients and, despite the fact that they were dying, they were still eager to give their best efforts to provide care to the patients.

In sum, participants asserted the importance of making it known to the patients that someone was taking care of them. These efforts are seen as vital aspects within the theory of bureaucratic caring and the peaceful end of life theory. In addition, the participants considered doing everything they can, to the best of their abilities, as a means to mitigate any psychological effect of futile care to COVID-19 patients.

### 3.4. Theme IV: Staying Close Amidst the Reshaped Work Environment

This theme highlighted the cluster of statements that describe how the workplace environment at the ICU has dramatically changed since the pre-pandemic period. It includes aspects such as routine bedside care, challenges associated with performing standard procedures, and the difference between the care required by COVID-19 patients and the usual ICU patients. From the clusters of statements, four sub-themes were identified, namely: continuing unhampered quality care despite risks, enduring the compounding troubles on the field, acknowledging the diversity of COVID-19 cases, and surmounting the burdens and surges of workloads.

#### 3.4.1. Continuing Unhampered Quality Care despite Risks

During the pandemic, the participants demonstrated an inclination to serve despite their ambivalent feelings about maintaining continuity of care. According to Geoffrey, “*the only difference for me was that we wore protective clothing and infection control measures were heightened. However, when it comes to the provision of healthcare to COVID-19 patients who are critically ill; as far as I am concerned, the quality of care has not been compromised.*” During COVID-19, every patient should receive the highest standard of care, regardless of their condition, which is the primary goal of both theory of bureaucratic caring and the peaceful end of life theory. Additionally, Milo explained that nurses in the ICU continued to provide different types of support to patients throughout this pandemic: “*So, continue the care, especially if they are under your care, because they need both emotional and spiritual support.*” When ICUs are focused on the preservation of life, they may have the perception that death is synonymous with failure. Therefore, the tsunami of deaths caused by this pandemic may cause stress and distress [9]. Even so, the majority of participants remained in their respective healthcare institutions. In Serena’s words, “*despite our ambivalence, we still need to go there; we must be upfront and provide patients with the best possible care.*”

#### 3.4.2. Enduring the Compounding Troubles on the Field

This sub-theme noted the exacerbation of different challenges faced by participants as the pandemic progressed, aside from wearing PPE and being precise with their actions. Concerns about accidental exposure magnify the challenges associated with caring for dying COVID-19 patients. Milo stated that “*one of the most challenging aspects of COVID-19 is the prevention of accidental exposure through aerosol particles.*” In emergency situations, the importance of wearing complete PPE hindered the immediate response of ICU nurses. As Winifred explained: “*It is not easy to enter and exit the room. It is not possible to provide immediate care to the patient..., you must first protect yourself to avoid endangering others’ lives.*”

In addition, the lack of vital resources in both machines and medications has added to the burden on nurses [5]. Winifred stated: “*There was a time when the medication was unavailable. This made me feel extremely uncomfortable.*” Furthermore, nurses working in ICUs who were under intense pressure to continue working despite experiencing fatigue, burnout, and symptoms of the virus themselves were suffering unprecedented physical, psychological, and moral injuries due to the lack of knowledge regarding the course of treatment [27]. Frederick explained: “*So, it was a bit difficult at first, since we did not know what to do with them. After all, what is the correct name for this? There are limited resources... It’s a very challenging situation, and with a heavy heart, there are so few options available to you.*”

#### 3.4.3. Acknowledging the Diversity of COVID-19 Cases

In the ICU, COVID-19 cases are quite different. This is in contrast to other chronic diseases, such as cancer, where the patient somehow understands where he or she is heading. Milo stated: “*COVID-19 is not the same as other illnesses, such as cancer patients in the fourth stage who have no cure, at least by that time, they know where they are heading. In contrast, among COVID-19 patients, it is as if the disease was an accident*.” It is because of this phenomenon that EoLC is quite different for a dying COVID-19 patient. To add, the transmissibility of the COVID-19 virus and the mandatory use of PPE have also contributed to the paradigm shift in critical care [36]. According to Olivia, “*PPE should always be worn. Prior to the pandemic, you could simply enter the patient’s room. Now it is different, you must protect yourself from COVID-19.*”

Participants also noted that witnessing the death of COVID-19 patients is quite different from witnessing the death of non-COVID-19 patients. According to Frederick, “*…most patients are intubated. It is possible that normal patients will eventually recover and then be extubated. For COVID-19 patients, they will not.*” Furthermore, the amount of time spent with COVID-19 patients is also quite limited, as stated in the guidelines. According to Solomon, “*I have been handling dying patients for the past 12 years in the ICU, so it is not that new to me. The only difference is that this is COVID-19, and it is contagious.*”

#### 3.4.4. Surmounting from the Burden and Surge of Workload

This sub-theme described how participants cope with the heavy burden of treating critically ill COVID-19 patients as well as ensuring a comfortable and peaceful death for those with poor prognoses or those on DNR status. Participants believed that sharing the load greatly reduced their stress levels, which is a common experience for COVID-19 nurses [34,35]. They find opportunities to express their feelings, their compassion and pity towards the patient, as well as their frustrations and problems. As Winifred explained: “*Sometimes, I speak with my colleagues or feel the need to debrief myself with my supervisor. There are also some small gatherings where we share our feelings. It is critical to check our feelings from time to time.*” Olivia also provided an example of how she handled her negative emotions. She stated, “*Yes, sharing stories is like venting out. I don’t have to carry everything, the heavy burden transferred to me by the patient, at least I can share them with those who can relate.*”

Another method for reducing workload is through time management. Due to the fact that this is a novel condition and they have limited knowledge and training, all they can do for the dying patient is to do their duty. As Geoffrey reiterated: “*True, if the patient dies, we might also feel upset because it is a life lost, but there is also a sense that we tried our very best.*” Participants also indicated that doing the things they love assisted them in coping with the demands of providing EoLC in COVID-19 ward. Winifred noted that: “*As an individual, I still resorted to doing things that I love, like when I get home, I watch TV and relax myself and leave my feelings at work.*”

ICU nurses are dynamic in nature and the impact of COVID-19 on their workload is often considered passive, including unplanned and under-resourced changes. Therefore, nurses must be able to cope with the challenges brought by pandemics, which include protective measures, faith-based practices, social support, and psychological support [55]. Collectively, these resources work hand-in-hand to deliver quality EoLC.

### 3.5. Theme V: Preparing the Family Life after a Loved One’s Departure

Families of dying COVID-19 patients have often expressed concern about facing death, as being with a dying loved one or person can be a frightening experience. ICU nurses should assess and prepare for the needs of loved ones through careful and clear communication [38]. As EoLC is being provided, there is already an element of inclusivity on the part of the family members, as the COVID-19 ICU nurses are also addressing their emotional and mental needs. Care for dying patients often involves the consideration of family members. According to the participants, family members may exhibit various emotions, including regret, guilt, anger, sadness, and uncertainty. As Axel related, the mother of a patient suffered a dreadful experience: “*She wept a lot, and I assured her that the medical team would do everything possible to take care of her son*.”

Additionally, the participants shared that providing support to family members was also a priority. As Frederick mentioned: “*Supporting the family and preparing them for the possible outcome of the patient is very critical.*” Furthermore, because protocols were being implemented, they were not permitted to physically be present in the ICU. Within the context of caring for the family of a dying patient, fulfilling the family’s emotional needs is also a part of the process. As Solomon explained, “*I usually include the family in the nursing care that I provide due to the difficulty involved. As it is difficult to think about your family member dying from COVID-19, there is the issue of immediate cremation or burial. It is really very difficult to imagine that.*” The participants’ experiences in caring for the patients’ love ones are revealed in four sub-themes, including: respecting difficult informed decisions, bearing unfavorable information about the course of death, bridging the gap through technology, and journeying with the bereaved in the grieving process.

#### 3.5.1. Respecting Difficult Informed Decisions

This sub-theme is about close communication and the facilitation of doctor-family conferences that enable them to make difficult informed decisions. It is pertinent to note that each family has its own specific reasons for acting aggressively. In addition, each family may decide to wait until the patient passes away on his or her own. Besides the patient’s poor prognosis, financial considerations are also taken into account along with his or her co-morbidities and age. When a DNR decision is made in a particular medical situation, ethical dilemmas may arise for the patient and family, healthcare providers, including nurses, and the institution [56]. Axel reiterated, “*When they sign the DNR form, it is expected that you have presented them with all the information they require. In other words, I am referring to all the scientific evidence, including X-ray images, that can be viewed on a cellphone.*”

The participants emphasized the importance of signing the informed consent for DNR or treatment refusal. In some instances, one of the reasons for making sure the family is well informed is to enable them to be guilt-free following the death of the patient. The participants, however, believe that no matter what, this decision is quite distressing. According to Serena, “*…signing a DNR form can be very frightening and distressing for a family. This is particularly true when a loved one’s life is being taken away from them. There is a great deal of difficulty involved and Yes, it is extremely difficult to witness.*”

#### 3.5.2. Bearing Unfavorable Information about the Course of Death

This sub-theme is associated with the statements that depict the experience relative to the provision of appraisal regarding the patient’s current status. Working as an ICU nurse for COVID-19 patients, participants observed how the disease is progressing and how COVID-19 gradually results in the patient’s death. Usually, this occurs when the patient already has a poor prognosis. As Callum stated, “*The first thing we do for our patients on DNR status is to brief the family members on the patient’s condition.*” The participants also agreed that this intervention was similar to providing information and health education to the relatives of their patients. As Olivia stated, “*Health education is really applicable to cases in which additional information is given to patients’ love ones rather than to the patients themselves.*”

Due to the fact that the participants are usually alone in the room with the patient, the family members are likely to rely on the assessment provided by the nurses in the ICU. As a result of this collaborative effort, the physician will be able to formulate a prognosis before meeting with the patient’s family members. According to Milo, “*we must also focus on the family. Most of the time, the family members also have many questions. Since they were unable to see the patient, what they can do is rely to on us.*” Providing support to family members is of the utmost importance. The best way to address their anxiety is to let them be heard and to allow them to express their concerns. A helpful step for family members is to provide them with the structure to know what to expect, including the schedule of routines, the type of treatment being administered, and how their dying loved one is being cared for and comforted.

#### 3.5.3. Bridging the Gap through Technology

This sub-theme addressed the facilitation of communication and updates between patients, their loved ones, and physicians through the use of technology. Among these technologies are video calls and cellular phone calls. The use of modern telecommunications technology can play an instrumental role in the care of COVID-19 patients [57]. The participants reported that isolation made it difficult for family members to communicate with their patient. As Winifred shared, “*During end of life situations, the patient cannot be seen, so proper communication with the relatives is difficult due to the isolation that occurs. So we facilitate video calls and audio calls.*” Communication is so vital that ICU nurses will extend their time and take risks to facilitate the conversation between the patient and their loved one. Milo shared, “*Sometimes we have to stay longer inside the patient’s room because we have to hold the phone while their family is talking to them.*”

Within the ICU, patients are generally alone in the room. Listening to or seeing their loved ones can really uplift them and provide them with emotional and social support. As Callum mentioned, “*... sometimes relatives need to contact their father, or mother, they call, even just by hearing them is okay. This is a part of the emotional support.*” During EoLC, it is extremely important for patients and family members to be able to see one another for the last time; even by means of a virtual platform. Callum further explained, “*So for family interaction, those who are quarantined and can’t go out. What we do is to have them connect online and talk with the patient. As long as they can communicate with their family members before the end of life.*”

As the COVID-19 patient is already dying, the participants agreed that the needs of the family members to see the patient for the final time and bid their last farewells, for humanitarian reasons, take precedence over the ICU protocol. Olivia stated, “*Sometimes, I violate the confidentiality and protocols of the hospital since, for example, we are not permitted to take pictures or videos within the ICU. It is, however, not uncommon for the folks to request the last time they see their parents or siblings if the patient is dying. So there were times that we allowed with instruction not to record the video or no picture would be taken while online.*”

In addition, the participants expressed the desire to use technology to facilitate online chaplain, religious, or spiritual services for the patient for the final time. Depending on the patient’s religion, the service would differ. Irene noted that, “*... however, if they have family members who are not Roman Catholic, if they wish to hear a prayer, we could still put their cellphones on their ears.*” Lastly, the participants also expressed their role in facilitating communication between doctors, family members or the patients to reduce skepticism. Serena explained, “*We need to have the different departments or specialized doctors to talk to each other about how to coordinate their care for their patients and also to inform the families about their care to allay their skepticism. Because again, we are actually not really that familiar with the management of COVID-19, so updating is always needed.*”

#### 3.5.4. Journeying with the Bereaved in the Grieving Process

In this last sub-theme, participants shared their experiences with the family members as they embarked on the grieving process following the death of the COVID-19 patient. According to Milo, “*If they need someone to talk to for the grieving process, if you will not be affected, or if there will be no breach of protocol, just stay with them. Our responsibility is to provide comfort to the family.*” During the interview, participants noted that some family members were in denial regarding the cause of death. The primary role of the ICU nurses was to help them accept the death of their loved one by providing detailed information and facilitating a conference with the physician. Hence, regular updates should be provided in order to prevent situations of unacceptance and complaints. One of the most difficult trials of life is the loss of a loved one. However, most people are able to weather these storms with the support and comfort of their loved ones.

Many participants described how difficult it was to witness the patients die alone without being able to be with their family and love ones. Winifred stated, “*It’s a bit painful to witness the family see their loved ones for the last time. It was not possible for them to hold them physically; it was impossible for them to say goodbye properly; they could not hug a departing individual physically. There is a great deal of crying on their part. In front of the family, we maintain professionalism regardless of their feelings towards me. It was the same every day when I returned home. Nevertheless, it was still manageable.*”

As the nurses begin the journey with the patient and family, they may spend time with them and seek their perspectives and understanding of the patient’s condition. Family members may also require spiritual and emotional support by offering them spiritual advisors or involving them in the pastoral care of the patient through virtual anointing prayers or religious rituals that are in keeping with the patient’s faith and beliefs [58]. Providing bereavement care to family members following the death of a patient in the ICU has been shown to reduce the risk of post-traumatic stress disorder and prolonged grief [59].

In sum, following up with family members is highly appreciated by them. Family members can explore and identify the events that led to the death of their loved one during such a meeting, which may assist them in coming to terms with their loss. Furthermore, participants stated that respecting the family members’ privacy and giving them time to grieve are essential aspects of caring for them after the loss of a dear one. When a loved one passes away, it is a significant change in the family’s lives, and the time spent with them is crucial to the grieving process. Within the theory of bureaucratic caring, it is important to understand how people are connected [20], Therefore, it is important to understand and provide time for the grieving process.

## 4. Conclusions

The current study examined the lived experiences of ICU nurses providing EoLC to critically ill COVID-19 patients with poor prognoses or in DNR status. A number of limitations were identified in the study. As a result of the recent emergence of COVID-19 cases within the local community, communications and consent acquisition were conducted via Facebook Messenger or email, and interviews were conducted using the online platform Zoom. Observations of gestures were limited to the upper part of the body due to the angle at which the camera was pointed at the participants. Furthermore, the current study concentrates on nurses working in hospital settings within the Western Philippines, which may not be representative of the national capital region, where resource availability may vary. 

Using both the theory of bureaucratic caring and the peaceful end of life theory as a framework, it is evident from the lived experience of the COVID-19 ICU nurses that they have a clear understanding of their role as they face risky encounters in the course of their duties. An obvious difference between the provision of EoLC before and during the pandemic was the presence of ambivalent feelings and dilemmas that forced ICU nurses to choose between self-preservation and precarious interventions. Nevertheless, some participants reported that despite this sense of concern about being infected with COVID-19, they still carried out emergency operations while wearing only level 2 PPE. It was also clear to the participants that one of their primary responsibilities at the bedside as the primary care provider is to identify signs of impending death. This is in order to inform physicians and family members as soon as possible. As a result of these risky encounters, the participants explained that the negative emotions they experienced while working were well known to them. 

As a result, the phenomenon of EoLC persists among dying COVID-19 patients despite the lack of a protocol. Even with altered working methods, an increase in workload, elevated stress, anxiety, and fear due to the contagious disease conditions, and mixed feelings of compassion and fear, the participants were still able to incorporate the core principle the dying COVID-19 patient hopes to experience, which agrees with both of the aforementioned theories. Moreover, in order to make the peaceful end of life theory a reality, the acceptance of mortality and the end of life, free of suffering through symptoms management, feelings of dignity and respect, as well as the presence of loved ones surrounding comfort with the use of video calls and virtual platforms. Additionally, COVID-19 ICU nurses are required to maintain continuity of holistic care while maintaining personal safety and self-preservation goals, to use PPE, and to modify routine care with the integration of digital spiritual and family support.

### Recommendations

A major challenge for hospital administrators is to collaborate and develop unified policies and protocols regarding the provision of EoLC to dying patients. Funding is also needed to purchase equipment, including digital devices, music therapy gadgets, systems, and infrastructure that cater to the holistic needs of patients. The provision of an online service may be a suitable alternative if the need for physical interaction cannot be met. An isolation room with cameras can be established to enable feasible and real-time monitoring of patients, thus reducing the need for PPE and unnecessary room visits that may expose nurses and physicians to contaminants.

The hospital administration should also establish a highly specialized team for the provision of EoLC, including training in therapeutic communication, psychological first aid, and bereavement support. It is important that this team includes nurses not only from the ICU, but also from other departments that may treat infectious patients in the future. Aside from training the staff nurses, the hospital administrator should set up a mental health department. This will assist the nurses in caring for the bereaved family; particularly for those who are in denial or who have shown signs of distress. Psychological support may also be provided for nurses who are experiencing difficulties. 

For ICU nurses, it is imperative that they are aware of the various core concepts involved in EoLC. To ensure the safety of their patients, they must make prudent decisions regarding vital matters. In order to have guidance and direction when they are assigned to a dying COVID-19 patient, ICU nurses should be proactive in requesting and collaborating with the administration in the establishment of EoLC protocols in hospitals without one. PPE must be worn to ensure that they can provide holistic care while maintaining their own safety. Through the establishment of a protocol, patients dying of COVID-19 and other infectious diseases will be treated with high-quality unified care which will result in quality EoLC.

Importantly, when appropriate policies or protocols are put in place and correctly followed, ICU nurses do not face the fear of being discriminated against by patients, family members or other healthcare professionals. As a result, nurses will be provided with additional legal protection. It is also important for ICU nurses to be proactive in seeking professional help from a mental health expert if they experience any negative feelings or thoughts as a result of providing EoLCs. Furthermore, hospital administrations should provide nurses with training in mental tenacity and resilience.

Finally, it may be necessary to conduct further research to determine the extent to which COVID-19 ICU nurses are aware of, have an attitude toward, and adhere to EoLC concepts. It may also be recommended that further research be carried out with other ICU nurses who hold different religious or spiritual beliefs. This will enable us to determine how their beliefs may influence the provision of EoLC. It is also possible to conduct studies to assess the awareness and attitude of the general public towards advance directives and EoLC.

## Figures and Tables

**Table 1:** Participants of the study.

Name	Age	Gender	Total-Service ^1^	ICU-Service ^1^	COV-Service ^2^	Hospital
Milo	32	Male	5	5	7 Ms	Private
Olivia	31	Female	4	3	2 Ms	Private
Callum	33	Male	12	5	1 Y & 10 Ms	Private
Axel	31	Male	10	5	8 Ms	Private
Winifred	24	Male	4	3	1 Y & 11 Ms	Private
Frederick	32	Male	11	7	5 Ms	Public
Shiloh	43	Female	10	9	2 Ys	Public
Serena	35	Female	10	8	1 Y	Public
Solomon	44	Male	14	12	1 M	Private
Siegfried	31	Male	8	8	2 Ys	Private
Irene	37	Female	9	4	6 Ms	Private
Geoffrey	35	Male	15	10	9 Ms	Public

^1^ Service is in years. ^2^ Y signifies year(s), while M signifies month(s) of service in COVID-19 ICU unit.

## Data Availability

Data for the current study are not publicly available due to privacy concerns.
